# Supplementary material for: Association of Regional Bone Synthetic Activities of Vertebral Corners and Vertebral Bodies Quantified Using 18F-Fluoride Positron Emission Tomography with Bone Mineral Density on Dual Energy X-ray Absorptiometry in Patients with Ankylosing Spondylitis
Source: J Clin Med. 2020 Aug 17;9(8):2656. doi: 10.3390/jcm9082656 (PMC7465964; doi:10.3390/jcm9082656)
Supplement: Supplementary file 1 [file jcm-09-02656-s001.pdf]

Supplementary Table 1. The 18F-fluoride maximal standardized uptake value of 48 lumbar vertebrae .

	Lumbar vertebrae (*n* = 48)
^{18}F-Fluoride SUVmax of vertebral corner[a], mean ± SD	6.75 ± 1.81
^{18}F-fluoride SUVmax of vertebral body, mean ± SD	5.46 ± 1.97

[a] This indicates the arithmetic mean SUVmax of the upper and lower vertebral corners.

SUVmax: maximal standardized uptake values, BMD: bone mineral density

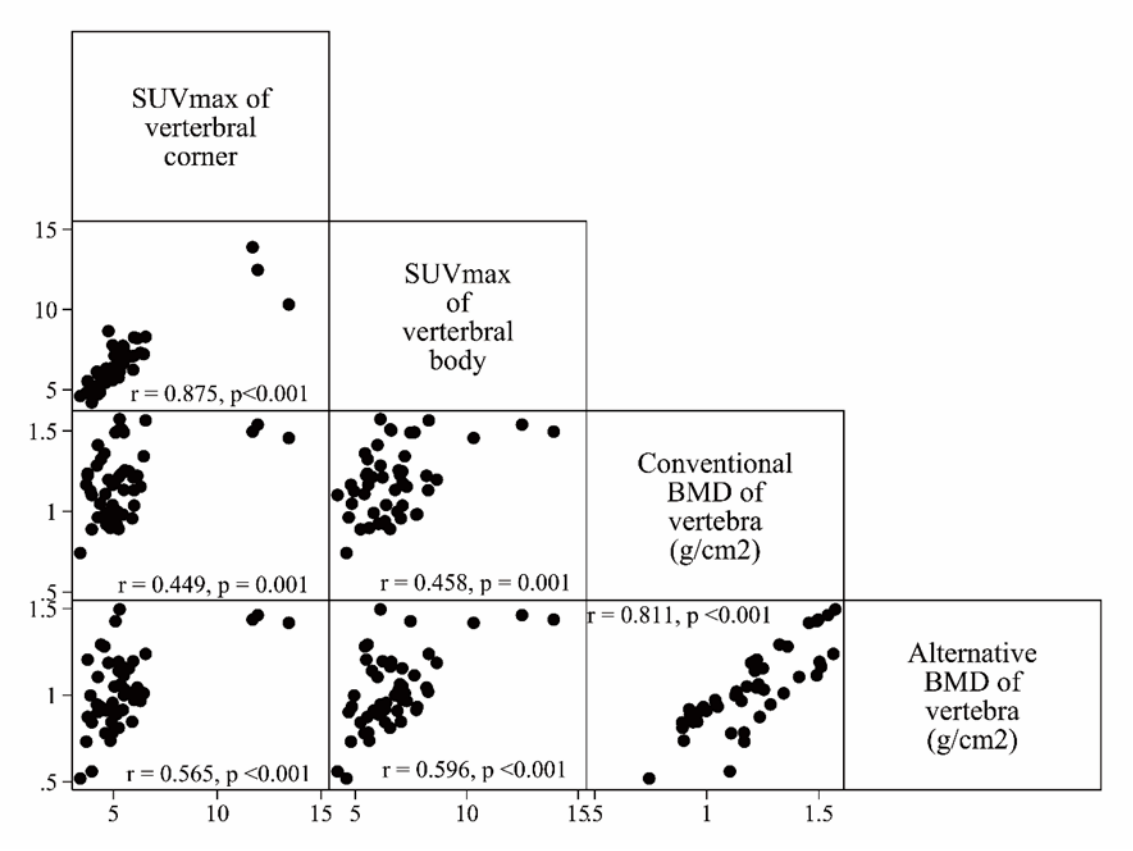

Supplementary Figure 1. Correlation plots between baseline 18F-fluoride maximal standardized uptake values of the vertebral bodies and corners and conventional and alternative bone mineral density at each vertebra level.

Supplementary Table 2. Relationship between bone mineral density and 18F-fluoride maximal standardized uptake value of each vertebrae analyzed by univariable generalized estimating equations adjusting for within patients correlations for the total number of lumbar vertebrae

Dependent variable	Independent variable	Univariable model	
		Unstandardized β (SE)	*p* value
Conventional BMD of vertebra (g/cm^2)	^{18}F-fluoride SUVmax of vertebral corner[a]	0.043 (0.012)	0.001
Conventional BMD of vertebra (g/cm^2)	^{18}F-fluoride SUVmax of vertebral body	0.051 (0.014)	<0.001
Alternative BMD of vertebra (g/cm^2)	^{18}F-fluoride SUVmax of vertebral corner[a]	0.054 (0.013)	<0.001
Alternative BMD of vertebra (g/cm^2)	^{18}F-fluoride SUVmax of vertebral body	0.065 (0.015)	<0.001
^{18}F-fluoride SUVmax of vertebral corner[a]	^{18}F-fluoride SUVmax of vertebral body	0.947 (0.08)	<0.001

[a] This indicates the arithmetic mean SUVmax of the upper and lower vertebral corners.

SUVmax: maximal standardized uptake values, BMD: bone mineral density.

Supplementary Table 3. Association between 18F-fluoride maximal standardized uptake value and bone mineral density at each lumbar vertebra level assessed by generalized estimating equations adjusting for within patients correlations for the total number of vertebrae

Dependent variable	Independent variable	Univariable model		Multivariable model[b]	
		Unstandardized β (SE)	*p* value	Unstandardized β (SE)	*p* value
Conventional BMD (g/cm^2)	^{18}F-fluoride SUVmax of vertebral corner[a]	0.043 (0.012)	0.001	0.008 (0.021)	0.724
	^{18}F-fluoride SUVmax of vertebral body	0.051 (0.014)	<0.001	0.046 (0.024)	0.055
	Disease duration (months)	0.002 (0.001)	0.014	0.002 (0.001)	0.019
	CRP (mg/dL)	-0.153 (0.096)	0.111	-0.129 (0.067)	0.06
	Age (years)	-0.001 (0.006)	0.84		
	BMI (kg/m^2)	0.013 (0.012)	0.294		
	BASDAI	-0.017 (0.021)	0.426		
Alternative BMD (g/cm^2)	^{18}F-fluoride SUVmax of vertebral corner[a]	0.054 (0.013)	<0.001	0.023 (0.024)	0.325
	^{18}F-fluoride SUVmax of vertebral body	0.065 (0.015)	<0.001	0.04 (0.027)	0.137
	BMI (kg/m^2)	0.017 (0.012)	0.148	0.01 (0.009)	0.262
	Disease duration (months)	0.001 (0.001)	0.549		
	CRP (mg/dL)	-0.067 (0.104)	0.518		
	Age (years)	-0.006 (0.006)	0.305		
	BASDAI	-0.004 (0.022)	0.866		

[a] This indicates the arithmetic mean SUVmax of the upper and lower vertebral corners.

[b]Estimated using multivariable generalized estimating equation models including ^{18}F-fluoride SUVmax of vertebral body and vertebral corners, disease duration and CRP

for conventional BMD and including ^{18}F-fluoride SUVmax of vertebral body and vertebral corners and BMI for alternative BMD, respectively.

SE: standard error, BMD: bone mineral density, SUVmax: maximal standardized uptake values, CRP: C-reactive protein, BMI: body mass index, BASDAI: Bath Ankylosing Spondylitis Disease Activity Index.
